# Impact of Vision Defects on Tooth Shade Selection: A Comparative of Spectrophotometry and Shade Guides in a Cross-Sectional Clinical Study

**DOI:** 10.3390/jcm14010293

**Published:** 2025-01-06

**Authors:** Mario Alvarado-Lorenzo, Eva Lozano-Garcia, Pedro Colino-Gallardo, Luis Daniel Pellicer Castillo, Victor Díaz-Flores García, Alfonso Alvarado-Lorenzo

**Affiliations:** 1 Department of Dentistry, Universidad Católica San Antonio de Murcia, 30107 Murcia, Spain; malvarado@pgoucam.com (M.A.-L.); peri.colino@pgoucam.com (P.C.-G.); 2 Department of Health Science, Miguel de Cervantes European University of Valladolid, 47012 Valladolid, Spain; elozano@uemc.es (E.L.-G.); ldpellicer@uemc.es (L.D.P.C.); 3 Department of Clínical Denstistry, University European of Madrid, Madrid, 28670 Vilaviciosa de Odón, Spain; 4 Department of Oral Surgery, Universidad de Salamanca, 37007 Salamanca, Spain; kuki@usal.es

**Keywords:** colour measurement, refractive errors, spectrophotometer, dental colourimetry, visual alterations

## Abstract

**Introduction:** Tooth shade selection is a fundamental factor in the success of dental restorations, and visual impairment may adversely affect this process. The aim of this cross-sectional clinical study was to determine whether visual impairment influences shade selection using two methods: spectrophotometry and shade guides. **Materials and Methods**: The sample consisted of 2796 maxillary and mandibular teeth, and shade selection was measured subjectively with a shade guide (VITA Classic, VITA Zahnfabrik) and objectively with a spectrophotometer (VITA Easyshade^®^ V, VITA Zahnfabrik, Bad Säckingen, Germany). In all cases, three measurements were taken on each tooth, with a waiting time of 15 min between samples. Shade selection was compared between observers with normal vision, myopia, astigmatism, and hyperopia. **Results**: The results show that myopic subjects perceived the lower central incisors (2.63, *p* < 0.05), upper lateral incisors (2.42, *p* < 0.05), lower lateral incisors (2.34, *p* < 0.05), and lower canines (2.64, *p* < 0.05) more clearly. Non-astigmatic subjects perceived the lower second premolar as lighter than astigmatic subjects (−2.01, *p* < 0.05). **Conclusions:** It can be concluded that myopes see teeth more clearly, but no differences have been found in astigmatism and hyperopes.

## 1. Introduction

One of the major challenges in aesthetic dentistry today is to match the shape and shade of restoration to the contralateral tooth without restorations [1,2,3]. The human perception of colour is based on a combination of three elements: the properties of the light striking the object, the chemical properties of the object that determine how it absorbs or reflects light, and the human visual system [4].

There are intrinsic properties of the tooth that affect its colour, such as translucency, opalescence, fluorescence and metamerism [3,5,6,7,8,9]. There are also extrinsic factors that influence tooth colour, such as visual fatigue, the age of the professional taking the shade [10,11], and the quality and quantity of the light source, which must be ambient light as it contains all visible wavelengths [12,13].

The most common method of shade-taking in dental practises is to use shade guides for comparison, known as subjective colourimetry [1]. One of the most widely used guides is the VITA Classic, which organises the shade guides into shade groups. However, this guide does not represent all possible tooth shades [2,14]. Subsequently, the VITA 3D-Master guide was developed, which is divided into five groups according to brightness and facilitates shade selection due to its greater coverage and uniform distribution of shades [15].

Science is advancing, and new technologies have been developed to make shade-taking more successful, treatments more predictable, and improve communication with the laboratory. This helps to avoid the factors involved in subjective shade-taking, although a combination of both shade-taking techniques can also be used [1,3,16]. Of all the instruments used to measure colour, the spectrophotometer, known as objective colourimetry, is the most accurate [17]. To measure tooth colour, it measures the amount of light reflected from the tooth [18]. The spectrophotometer consists of a light scattering device, an optical device and a detector that converts the reflected light into a signal that is then translated into colour information for the dentist [19]. Although this technique is expensive and requires training to use, it offers advantages such as reduced patient time in the dental office and objectivity in shade assessment [17,20,21].

Refractive errors include myopia, hyperopia, and astigmatism. Myopia is characterised by difficulty focusing on distant objects, although objects close to the observer are seen in focus on the retina without the need for accommodation [22]. In hyperopia, the image of a near object appears blurred when the observer is relaxed. This can sometimes cause headaches, double vision, or redness of the eyes in low light or after focusing on a close object for a long time [23]. With astigmatism, objects appear stretched and distorted. Those with no refractive error are considered to be emmetropic [22,23].

The influence of visual defects on subjective and objective colourimetry in dentistry has been analysed in the scientific literature, although the results are not conclusive [9,13,14,15,24,25]. In the study published by Khosla et al., which analysed the influence of vision impairments on colour selection, it was concluded that there were no significant differences compared to the normal vision group with subjective colourimetry using dental guides [25]. However, when comparing not only vision defects but also subjective colour perception with dental guides and objective colour perception with measuring devices such as spectrophotometers, digital photography, and even smartphones, studies have found that subjective colour guidance is less reliable and reproducible. Furthermore, it is also conditioned by environmental factors such as the type of lighting and observer fatigue [26]. Additionally, systemic disease factors affecting vision, such as type-1 diabetes, can negatively affect dental shade perception [27].

In the study by Pohlen et al., the authors analyse the impact of varying degrees of chromatic vision impairment on dental shade selection. Their findings indicate that patients with greater chromatic deficiency tend to exhibit poorer shade selection in dental guides, which has a negative effect [28]. However, there is a paucity of research in the scientific literature on the subject of refractive errors in vision, such as myopia, astigmatism, and hyperopia. This study, therefore, aims to address this gap in the literature.

Therefore, the aim of the present study is to analyse the effect of visual impairment on dental colourimetry using dental guides and spectrophotometry. The null hypothesis of this clinical study is that visual impairment does not affect dental colourimetry using dental guides and spectrophotometry in terms of brightness or luminosity.

## 2. Materials and Methods

### 2.1. Study Design

This study was approved by the Valladolid Health Area Drug Research Ethics Committee, with registration number PI 20-1911-2020, and the study followed the ethical guidelines of the Declaration of Helsinki for biomedical research. A cross-sectional clinical study was carried out in which 2768 natural teeth were measured in 294 patients. Participants were informed of the object of the study, and their informed consent for participation was obtained. The shade was measured objectively with the VITA Easyshade^®^ V spectrophotometer (VITA Zahnfabrik, Germany) and subjectively with the VITA Classical A1-D4 shade guides (VITA Zahnfabrik, Germany). Previously published studies were taken into account when determining the sample size [29,30,31,32], which had smaller sample sizes.

### 2.2. Patient Selection

Of the 294 Spanish Caucasian patients, where the age of men was 36.5 years, 154 were female, and 140 were male. Inclusion criteria for the study subjects were patients with young permanent dentition, preferably with the following natural teeth: upper and lower left and right central incisors, lateral incisors, canines, first bicuspids, first premolars, and second bicuspids. Exclusion criteria were teeth with restorations, veneers or crowns, endodontic teeth, teeth with orthodontic retention on the palatal side, bleached teeth, or teeth that could not be coloured.

**Figure 1:**
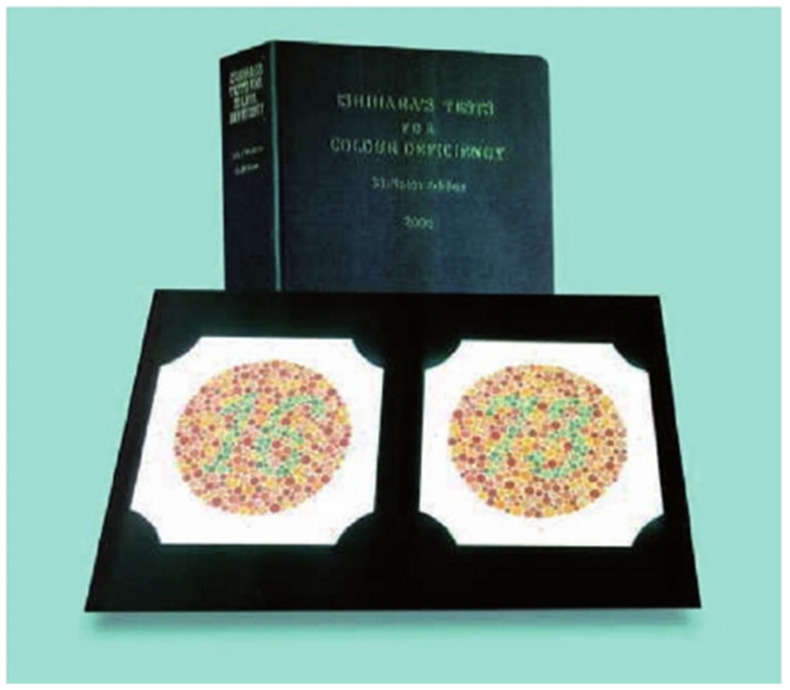
Ishihara test.

Each was measured by a final-year dental student at the European University of Valladolid, of whom 171 were female and 123 were male. The students ranged in age from 21 to 51 years, with a mean age of 24.48 years. To determine the refractive errors, an ophthalmological examination was carried out on all the students who were instructed in the science of colour in dentistry and colourimetry prior to colour sampling. Students with dyschromatopsia were excluded, and the Ishihara test was performed (Figure 1). Of the total number of observers, 154 wore spectacles, and 140 did not. The most common refractive error in the sample was myopia, followed by astigmatism and hyperopia (Table 1). Of the total sample, 91 of the 294 observers did not require spectacles. Therefore, students with severe colour vision problems such as colour blindness, partial blindness in both eyes, systemic problems, such as type-1 diabetes with ocular involvement, and ocular degeneration problems such as cataract formation, were excluded from the study.

The descriptive results of the sample indicate that 58.2% were women compared to 41.8% men. Myopia was the most common visual disturbance, accounting for 48.8%, followed by astigmatism at 29.6%, and finally, hyperopia at 7% (Table 1).

### 2.3. Measurement Process

**Figure 2:**
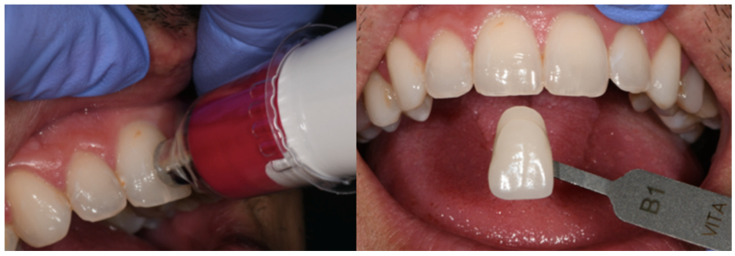
Objective and subjective colour measurements.

The colour measurement was carried out in an 8 m^2^ cabinet illuminated by both artificial light and natural light through two windows, each 1 metre wide and 1.5 metres high. Measurements were taken inside the cabinet using a photometer to identify areas of approximately 5500 degrees Kelvin to ensure correct illumination for the colour measurement process. The photometer used was the Sekonic Dual Spot L-778 (Sekonic Co., Tokyo, Japan). For the subjective colour assessment, the colour was selected using the VITA Classic guide. The guide was held 25–30 mm from the patient’s tooth, and the shade (colour) that most closely resembled the patient’s tooth was selected first. Then, the saturation (amount of grey in a shade) and value (amount of black or white in a shade) corresponding to the tooth under investigation were selected from the strips in that group. For the objective colour measurement using the VITA EasyShade spectrophotometer, the tooth must be hydrated, the tip of the spectrophotometer must be perpendicular to the vestibular side of the tooth being analysed, and it must remain stationary until the colour analysis results are obtained (Figure 2).

For both subjective and objective colourimetry, three measurements were taken on each tooth by each observer. There was a fifteen-minute waiting period between measurements. Values that agreed across the three measurements were recorded on the data collection sheet.

In order to be able to analyse the data in terms of the brightness of the classical vita-guide, it was arranged in ordinal values according to the following ordinal number order: B1 (15), A1 (14), A2 (13), D2 (12), B2 (11), C1 (10), C2 (9), D4 (8), D3 (7), A3 (6), B3 (5), A3,5 (4), B4 (3), C3 (2), A4 (1).

### 2.4. Statistical Analysis

The statistical analysis involved entering the research parameters into an Excel spreadsheet (Microsoft, Redmond, WA, USA) and performing the statistical analysis using SPSS V29.0 (IBM, Armonk, NY, USA). Methods such as the arithmetic mean, variance, standard deviation, and Chi-square test were used to derive the statistical data.

The study variables were dichotomous and categorical. Therefore, the Chi-square test (*p* < 0.05) was used to examine the relationship between correct judgments and the specific variables of each subject, such as myopia or astigmatism. Human judgments were compared with those obtained from spectrophotometers using Pearson’s correlation coefficient (*p* < 0.01).

## 3. Results

In Table 2, the correlations between the measurements of brightness made with the spectrophotometer and dental guides are presented, comparing the group with lenses and the group without lenses. In the group with lenses, the highest value was for the upper central incisor (0.65), and the lowest was for the lower second premolar (0.31), with *p* < 0.01. In the group without glasses, the highest value was for the upper canine (0.60), and the lowest was for the upper second premolar (0.37), with *p* < 0.01. The correlations are similar for both groups and in each group, the highest correlation is observed in the anterior sector, while the lowest is observed in the posterior or molar area.

An analysis was conducted to determine whether there were differences in the perceived means of clarity (classic guide) between lens wearers and non-users. Table 3 displays the comparison of the perceived means of clarity for both lens wearers and non-wearers. The greatest differences were observed in the anterior sector, with the upper lateral incisor showing the highest value (1.71, *p* > 0.05) and the lower second premolar showing the lowest value (−0.46, *p* > 0.05); however, these differences were not statistically significant.

The study investigated whether there were differences in the perceived means of clarity (classical guide) between myopes and non-myopes. The following table (Table 4) presents the results of the means and the level of significance. It is apparent from this sample that there are differences between myopes and non-myopes in perceived clarity. Myopes perceive greater clarity in the lower central incisors (2.63; *p* < 0.05), upper lateral incisors (2.42; *p* < 0.05), lower lateral incisors (2.34; *p* < 0.05), and lower canines (2.64; *p* < 0.05).

The study also investigated whether there were differences in the perceived means of clarity (classic guide) between astigmatism and non-astigmatism in the students. The following table (Table 5) presents the results of the means and their level of significance. Only one statistically significant mean difference was observed in relation to the lower second premolar. Non-astigmatic subjects perceived this tooth to be lighter compared to those with astigmatism (−2.01, *p* < 0.05).

It was analysed whether there were differences in the perceived means in clarity (the classic guide) for hyperopes and non-hyperopes. The following table (Table 6) shows the results of the means, where there are no significant differences between the two groups; however, there is a tendency for there to be a greater difference between the groups in the posterior sectors than in the anterior sectors except for the first lower premolar.

## 4. Discussion

This study analysed the influence of vision defects on dental colour acquisition, considering the colour acquisition performed objectively by a spectrophotometer to be valid. Several previous articles analysed the difference between objective and subjective shade-taking [33,34,35,36,37], and various studies involved dental students as the subjects [38,39,40]. The study observed that many errors occur when taking shade subjectively with dental guides, and several authors also argue that subjective shade-taking leads to more errors than an objective technique [33,34,35,36]. However, Parameswaran et al. consider combining visual methods with the spectrophotometer as ideal for shade determination [37].

In this study, the classical guide was used, and the illumination conditions were correct, as described previously, which is an important factor in shade selection with dental guides, as determined in the article by Śmielecka et al. [41]. They conclude that lighting conditions determine the reliability and reproducibility of the measurements.

Measurements were performed on final-year dental students. Students with colour deficiencies were excluded, and there was even a study concluding that the clinician’s personality also influences colour acquisition [42]. Statistically significant results were found in the study by Pohlen et al., although subjects with impaired colour vision performed worse when choosing shades during colour pick-up [27].

This study did not aim to determine whether training in dental colourimetry would enhance accuracy rates in subjective shade-taking; however, some authors assert that training in shade-taking is advantageous in achieving better results in tooth shade determination [32,40,41,43]. Jain et al. conducted their study on shade-taking with all dental undergraduate students and discovered that the students who participated were more accurate as they advanced to higher grades [40]. These findings are in contrast to those in the studies by Pohlen et al. and Udiljak et al., who argue that differences in subjective shade-taking success are not significant after attending a one-hour lecture on shade-taking and that the amount of clinical experience does not ensure that clinicians take the tooth shade more accurately [38,44].

The determination of clarity or brightness in the Vita classical guide was conducted in accordance with the article by Gómez-Polo et al. [45], in which they concluded that the dimension of colour with the greatest agreement between the operator and the spectrophotometer is the value or brightness. In Table 2, it can be observed that for the correlations between all colours with dental guides and spectrophotometry with lenses, there is greater agreement in the anterior teeth than in the posterior teeth; however, in the group without lenses, the agreement is similar and also lower. Therefore, whether the observer wears lenses or not is irrelevant to the measurement of tooth colour. In the article by Samra et al. [32], the influence of prior training in shade-taking with dental guides is discussed. They conclude that students with normal vision, myopia, and hyperopia improve their colour vision after education and training in colour vision. In this study, all observers received prior training, and the largest differences were found in the measurements of the myopic and non-myopic groups in Table 4. Given that myopia is a common visual defect, patients with myopia may also have other defects, such as astigmatism. The differences in other defects, such as hyperopia, were smaller because the sample size and prevalence of this defect were also smaller.

As myopia, hyperopia, and astigmatism are refractive errors related to visual acuity in the present study, it was found that myopia is the phenomenon that most negatively affects the determination of tooth colour, as described in Table 4. Myopia is a refractive error that, in principle, affects vision in the distance and, therefore, a priori, it should not have the largest statistical difference compared to hyperopia and astigmatism. As the population of observers was young, they should also have no accommodation problems with distance vision. The effect may have been statistically significant because the parameter measured was luminance, and neither the hue nor saturation was taken into account. Therefore, this statistical difference should be evaluated clinically in further studies.

This study had certain limitations, including the use of additional measuring devices such as spectrophotometers, colourimeters, and scanners. Additionally, the sample could be expanded to include observers of varying ages and experience levels. However, previous studies have indicated that experience alone does not guarantee optimal shade selection with dental guides. These factors will be further investigated in future studies to ascertain potential sources of variation.

## 5. Conclusions

There is no difference in the selection of tooth colour in observers with or without lenses.

Among the visual defects, myopes see the front teeth more clearly than non-myopes. However, astigmatic and hypermetropic observers have no significant differences.

## Figures and Tables

**Table 1:** Distribution of participants according to gender, percentage of lens wear, and vision impairment.

		n	%
Gender	Men	123	41.8
	Women	171	58.2
Vision disorders	Myopia	139	48.8
	Astigmatism	86	29.6
	Hyperopia	20	7
Prescription lenses	With lenses	154	53.8
	Without lenses	140	46.2

**Table 2:** Correlations of lens and non-lens wearers per tooth measured with spectrophotometer and dental guides of brightness.

Tooth Type	With Lenses n = 154	Without Lenses n = 140
Upper central incisor	0.65 *	0.47 *
Lower central incisor	0.47 *	0.52 *
Upper lateral incisor	0.43 *	0.44 *
Lower lateral incisor	0.50 *	0.54 *
Upper canine	0.55 *	0.60 *
Lower canine	0.54 *	0.55 *
1st upper premolar	0.37 *	0.46 *
1st lower premolar	0.59 *	0.47 *
2nd upper premolar	0.36 *	0.37 *
2nd lower premolar	0.31 *	0.44 *

* Significant *p* < 0.01.

**Table 3:** Mean difference between lens wear (n = 154) and no lens wear (n = 140).

	Lenses	Mean	Student’s *t*-Test	Significance Level
Upper central incisor	Yes	12.35	1.41	*p* > 0.05
	No	11.85		
Lower central incisor	Yes	10.96	1.48	*p* > 0.05
	No	10.32		
Upper lateral incisor	Yes	11.46	1.71	*p* > 0.05
	No	10.84		
Lower lateral incisor	Yes	9.90	0.90	*p* > 0.05
	No	9.49		
Upper canine	Yes	7.09	1.57	*p* > 0.05
	No	6.65		
Lower canine	Yes	6.36	1.60	*p* > 0.05
	No	5.69		
1st upper premolar	Yes	8.57	0.68	*p* > 0.05
	No	8.28		
1st lower premolar	Yes	6.80	0.38	*p* > 0.05
	No	6.65		
2nd upper premolar	Yes	8.43	−0.49	*p* > 0.05
	No	8.65		
2nd lower premolar	Yes	6.68	−0.46	*p* > 0.05
	No	6.87		

Significant *p* < 0.05.

**Table 4:** Difference in averages for myopes and non-myopes.

Tooth Type	Myopic	Mean	Student’s *t*	Significance Level
Upper central incisor	Yes	12.48	1.88	*p* > 0.05
	No	11.82		
Lower central incisor	Yes	11.23	2.63	*p* < 0.05 *
	No	10.11		
Upper lateral incisor	Yes	11.66	2.42	*p* < 0.05 *
	No	10.81		
Lower lateral incisor	Yes	10.30	2.34	*p* < 0.05 *
	No	9.25		
Upper canine	Yes	7.34	1.77	*p* > 0.05
	No	6.59		
Lower canine	Yes	6.62	2.64	*p* < 0.05 *
	No	5.51		
1st upper premolar	Yes	8.73	1.18	*p* > 0.05
	No	8.20		
1st lower premolar	Yes	6.81	0.60	*p* > 0.05
	No	6.57		
2nd upper premolar	Yes	8.52	−0.31	*p* > 0.05
	No	8.66		
2nd lower premolar	Yes	6.68	−0.32	*p* > 0.05
	No	6.82		

* Significant *p* < 0.05.

**Table 5:** Mean differences for astigmatism and non-astigmatism in students.

Tooth Type	Astigmatism	Mean	Student’s *t*-Test	Significance Level
Upper central incisor	Yes	12.14	0.20	*p* > 0.05
	No	12.06		
Lower central incisor	Yes	10.68	0.09	*p* > 0.05
	No	10.64		
Upper lateral incisor	Yes	11.27	0.45	*p* > 0.05
	No	11.08		
Lower lateral incisor	Yes	9.67	−0.06	*p* > 0.05
	No	9.70		
Upper canine	Yes	6.99	0.31	*p* > 0.05
	No	6.84		
Lower canine	Yes	6.16	0.44	*p* > 0.05
	No	5.96		
1st upper premolar	Yes	8.21	−0.63	*p* > 0.05
	No	8.53		
1st lower premolar	Yes	6.40	−0.92	*p* > 0.05
	No	6.81		
2nd upper premolar	Yes	8.25	−0.82	*p* > 0.05
	No	8.65		
2nd lower premolar	Yes	6.09	−2.01	*p* < 0.05 *
	No	7.00		

* Significant *p* < 0.05.

**Table 6:** Differences between hyperopic and non-hyperopic averages.

Tooth Type	Hypermetropia	Mean	Student’s *t*-Test	Signification Level
Upper central incisor	Yes	12.65	0.86	*p* > 0.05
	No	12.06		
Lower central incisor	Yes	10.40	−0.33	*p* > 0.05
	No	10.67		
Upper lateral incisor	Yes	11.68	0.78	*p* > 0.05
	No	11.13		
Lower lateral incisor	Yes	9.95	0.30	*p* > 0.05
	No	9.69		
Upper canine	Yes	7.45	0.72	*p* > 0.05
	No	6.86		
Lower canine	Yes	6.15	0.17	*p* > 0.05
	No	6.01		
1st upper premolar	Yes	9.42	1.26	*p* > 0.05
	No	8.35		
1st lower premolar	Yes	6.84	0.22	*p* > 0.05
	No	6.67		
2nd upper premolar	Yes	9.55	1.31	*p* > 0.05
	No	8.46		
2nd lower premolar	Yes	7.42	0.95	*p* > 0.05

Significant *p* < 0.05.

## Data Availability

The data are only available on request due to privacy restrictions.
